# Use of the non-radioactive SUnSET method to detect decreased protein synthesis in proteasome inhibited Arabidopsis roots

**DOI:** 10.1186/s13007-016-0120-z

**Published:** 2016-03-16

**Authors:** Doug Van Hoewyk

**Affiliations:** Biology Department, Coastal Carolina University, Conway, SC 29526 USA; Ankara University, Biotechnology Institute, Tandoğan Campus, 06110 Ankara, Turkey

## Abstract

**Background:**

In eukaryotic cells, the proteasome maintains homeostasis by selectively degrading regulatory and misfolded proteins, and in doing so contributes to the amino acid pool. Inhibition of the proteasome in yeast and human cells decreases de novo protein synthesis. However, it is not know if proteasome inhibition in plants similarly suppresses protein synthesis. To address this gap in plant biology, protein synthesis in Arabidopsis roots was estimated using SUface SEnsing of Translation (SUnSET) techniques. This non-radioactive method has been validated in animal cells, but has not yet been applied to plants. The goal of this study was to investigate the suitability of SUnSET methodology to measure protein synthesis in plants, and to determine if proteasome inhibition decreases levels of newly synthesized proteins.

**Results:**

The SUnSET technique revealed that Arabidopsis plants treated with cycloheximide—an inhibitor of protein synthesis—severely decreased levels of newly synthesized proteins in root and shoot tissue, as detected on a Western Blot. Therefore, the non-radioactive method is suitable to detect changes in protein synthesis, and was subsequently used to monitor protein synthesis in proteasome-inhibited roots. The proteasome inhibitor MG132 decreased levels of newly synthesized proteins by 70–80 % after 4 and 16 h. Removal of MG132 from liquid media resulted in roots with increased levels of newly synthesized proteins compared to untreated plants, suggesting that recovery from proteasome inhibition results in elevated levels of protein synthesis. Additionally, SUnSET was used to detect a decrease in protein synthesis in the roots of plants subjected to salt stress or sulfur starvation.

**Conclusions:**

Proteasome inhibition has been shown to decrease protein synthesis in yeast and human cells, and this study now shows that MG132’s inhibitory effects also applies to plants. These data represent the first time that SUnSET has been used to measure protein synthesis in plants. The study demonstrates that SUnSET is a suitable and robust technique to measure protein synthesis in plants. The use of this non-radioactive method to gauge protein synthesis offers a fast, safe, and cost-effective alternative compared to traditional techniques that rely upon radioactive material. The method is likely to have broad applicability to different disciplines in plant biology.

## Background

The ubiquitin proteasome pathway (UPP) is found in all eukaryotic cells and functions by selecting ubiquitinated proteins for proteasomal degradation in the cytosol or nucleus [1]. Proteolysis of ubiquitinated proteins by the proteasome serves a variety of functions. The proteasome degrades many short-lived regulatory proteins, including those governing in cell division [2]. In plants, the UPP can also regulate nutrient status [3] and hormone signaling [4]. The proteasome also clears the cell of oxidized or misfolded proteins that result from stress, thereby preventing toxic protein aggregation. In addition to the removal of regulatory and damaged proteins, it is estimated that about 30 % of newly synthesized proteins might undergo rapid proteasomal degradation [5].

The degradation of proteins mediated by the UPP serves to maintain the amino acid pool, but this has only been established in non-plant models. Proteasome inhibition in nutrient-deprived human cells depleted the amino acid pool and decreased protein synthesis [6]. More recently, it was reported that amino acid depletion resulting from UPP impairment results in lethality in human cells, yeast, and Drosophila [7]. Predictably, amino acid shortage induced by the proteasome inhibitor MG132 rapidly decreases de novo protein synthesis. In mouse cells treated with MG132, protein synthesis decreased by 90 % after 6 h [8]. A nearly similar result, i.e. 80 % decrease in protein synthesis, was observed in yeast with impaired proteasome activity [7]. MG132 has also been reported to decrease protein synthesis in neurons [9] and myoblasts [10].

The consequences of UPP inhibition are well-characterized in yeast and mammalian cells, and in humans is associated with several neurodegenerative diseases [11]. In contrast, our understanding of the consequences of proteasome inhibition in plants lags behind that of yeast and humans. This is unfortunate, given the increasing evidence suggesting that plant proteasomes area susceptible to a variety of stressors. For example, some plant pathogens can release effectors that directly impair the proteasome’s catalytic core, which serves to promote pathogenic infection in plants [12]. Evidence is also mounting that abiotic stress impairs the UPP. Decreased proteasome activity in plants can be caused by heavy metals [13–15] and salt stress [16]. Selenite stress also impairs the UPP, which was associated with an accumulation of superoxide in Chlamydomonas [17] and *Brassica napus* [18]. Plants can respond to proteasome inhibition by initiating autophagy, including the degradation of inactive proteasomes [19]. Despite these recent findings, it still is not known if proteasome impairment in plants decreases de novo protein synthesis.

This study employed SUface SEnsing of Translation (SUnSET) techniques to determine if proteasome inhibition in plants decreases the rate of newly synthesized proteins. This non-radioactive technique was recently reviewed [20], and has been widely employed in human cell lines [21] and human tissue, including skeletal muscle [22] and the hippocampus [23]; however, SUnSET has not been demonstrated in plants. Briefly, this technique requires treatment and uptake of the antibiotic puromycin, a structural analogue of tyrosal-tRNA that contains a non-hydrolyzable bond between the tRNA and amino acid. Incorporation of puromycin into nascent polypeptides causes termination. Although high concentrations of puromycin is toxic because it can inactivate translation, at low concentrations it provides an accurate snapshot of protein synthesis without causing lethality. Levels of newly synthesized proteins containing puromycin and its analogs [24] can be measured by immunohistochemistry, fluorescence activated cell sorting (FACS), and confocal microscopy [24]. Alternatively, the newly synthesized polypeptides can be detected as low-molecular weight bands on a Western-blot using an anti-puromycin antibody, and less cost-prohibitive.

SUnSET has been used to estimate both increased and decreased levels of newly synthesized proteins. For example, SUnSET has been used to detect decreased protein synthesis in cells treated with cycloheximide and arsenite [21], known inhibitors of protein synthesis. Additionally, the technique has also been applied to monitor increased protein synthesis in muscle cells treated with insulin [22]. In both studies, rates of protein synthesis were validated with established radioactive techniques, and changes in protein synthesis were nearly identical when comparing the two methodologies. The goal of this study was two-fold. Initial experiments were conducted to determine if SUnSET is a suitable method to gauge changes in protein synthesis, as estimated by Western-blotting. Secondly, SUnSET was employed to decifer if proteasome inhibition decreases the pool of newly synthesized proteins in roots.

## Methods

### Growth conditions

Arabidopsis plants (ecotype Columbia) were germinated on soil, and on day 7 transferred to aerated Hoagland’s media. Plants were hydroponically grown for an additional 21 days in a growth chamber (150 μEinsteins, 16 h light/8 h dark cycle, 24 °C) before subjected to additional stress treatments. To inhibit the proteasome, plants were transferred to 50 mL of aerated Hoagland’s media containing 0.1 % DMSO with or without 50 μM MG132, a concentration that is known to impair the proteasome in roots [18]. Plants were treated with MG132 for 0, 4, and 16 h. To induce salt stress, plants were treated with 100 mM NaCl for 4 day. Plants subjected to sulfur starvation were also grown for an additional 4 day; sulfur deprivation was induced by replacing sulfate-salts in the Hoagland’s media with chloride salts.

### SUnSET method and electrophoresis

After stress treatments, Arabidopsis plants were transferred into a 50 mL test tube containing 20 mL of Hoagland’s media with or without puromycin. Puromycin is toxic and inhibits protein synthesis at high concentrations. Therefore, to detect protein synthesis in roots, plants were initially treated with 2 and 20 μM for 30 min. To estimate protein synthesis in shoots, plants were treated with 20 and 50 μM puromycin for 30 and 60 min. Roots and shoots were separated and washed 2× in distilled water. Plant material was subsequently ground in liquid nitrogen, and a clarified protein extract was obtained for electrophoresis.

Newly synthesized proteins using the SUnSET method were detected by immunoblotting. To detect the truncated proteins the arise from puromycin’s misincorporation into polypeptides, 20 μg of protein were loaded onto a 15 % SDS-PAGE gel and run under denaturing conditions. Proteins were transferred on a PVDF membrane. Prior to immunoblotting, membranes were stained with Ponceau Red to ensure an equal loading of proteins. Membranes were subsequently washed, blocked in milk, and incubated for 2 h with the puromycin antibody (PMY-2A4) purchased from the University of Iowa, USA [25]. The PMY-2A4 anitbody was used at a 1:1000 dilution. Newly synthesized proteins containing puromycin were detected using a secondary antibody conjugated to alkaline phosphatase (1:10,000 dilution for 45 min). Protein synthesis was estimated based on the intensity of immunoreactive bands using image-J.

To confirm that SUnSET was suitable to detect changes in protein synthesis in roots, plants were treated with either 1 or 20 μM cycloheximide, a potent inhibitor of protein synthesis. After a 4 h treatment, protein synthesis in root tissue was estimated using SUnSET as described above.

Accumulation of high molecular weight poly-ubiquitinated proteins was detected on an 8 % SDS-PAGE gel [26]. Cox2, a 30 kDa protein, was analyzed from the same membrane that detected polyubiquitinated proteins. Bip2 (luminal binding protein 2) is 72 kDa, and was analyzed from the same membrane that detected newly synthesized proteins. Both Cox2 and Bip2 were analyzed as previously described [18].

### Microscopy and amino acid analysis

Cell viability in the root tips of plants treated with or without MG132 was estimated as similarly performed [18]. Briefly, excised root tips were incubated for 15 min in 1 mL of 50 mM Tris buffer, pH 7.5, containing 20 μM fluorescein diacetate. Root tips were subsequently washed 5× in Tris buffer to remove the fluorescent probe. Fluorescence of fluorescein diacetate (optimal Ex495/Em515) is dependent upon cell membrane integrity, and was estimated using a FITC filter set.

Levels of total amino acids were analyzed using a modified ninhydrin method [27]. Glutamic acid was used to make a standard curve, and spectrophotometrically measured at A_570_. Statistical analyses (ANOVA) were performed using the Kleida-graph software package (Synergy Software).

## Results

**Fig. 1:**
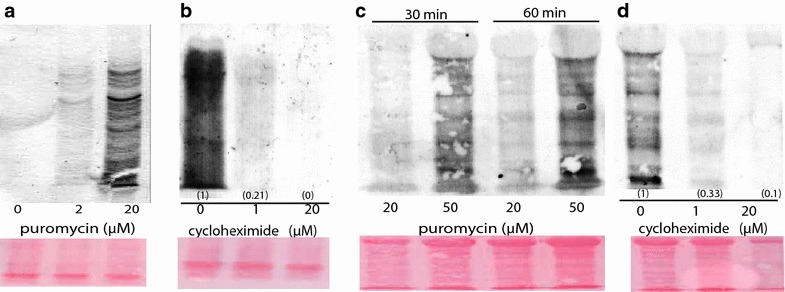
Detection of newly-synthesized proteins containing puromycin in root (**a**, **b**) and shoot (**c**, **d**) tissue. Cycloheximide treatment (4 h) decreases newly synthesized proteins in plants that were subsequently treated with 20 μM puromycin (**b**) or 50 μM puromycin (**d**). Plants were treated with puromycin for 30 min, except in (**c**). Twenty μg of protein were separated on a 15 % SDS-PAGE gel, and newly synthesized proteins containing puromycin immunoreacted against puromycin antiserum. Membranes were stained with Ponceau Red prior to immunblotting. *Smaller letters in parenthesis* represent band intensity relative to control on dried membranes using image-J. Images are representative of 3–4 experimental replicates

SUnSET has successfully been used to estimate changes in protein synthesis in yeast and animal cells, but the technique has not been employed in plants. Therefore, it was necessary to determine the suitability of SUnSET using appropriate controls. Hydroponically grown Arabidopsis plants were treated with puromycin (0, 2, and 20 μM) for 30 min, and proteins from root tissue were extracted. The intensity of the immunoreactive bands on a Western Blot correlated with puromycin concentration (Fig. 1a). Importantly, untreated control plants did not produce a band. Next, the effects of the cycloheximide—a protein synthesis inhibitor—were examined using SUnSET. Plants were treated with cycloheximide (0, 1, and 20 μM) for 4 h. After 4 h, roots treated with 1 μM cycloheximide exhibited an 80 % decrease in protein synthesis compared to untreated plants; newly synthesized proteins were not detected in Arabidopsis treated with 20 μM cycloheximide (Fig. 1b). Taken together, appearance of low molecular weight bands representing newly synthesized proteins were dependent upon puromycin treatment, but were largely absent during treatment with a protein synthesis inhibitor.

Although this study focused on root tissue, additional experiments sought to determine if SUnSET could also detect protein synthesis in leaves. Newly synthesized proteins could not be robustly detected in leaves when using the same conditions as for roots, i.e. 20 μM puromycin treatment for 30 min. However, newly synthesized proteins were visualized on a Western Blot when plants were treated with 50 μM puromycin for 30 and 60 min (Fig. 1c). To determine if SUnSET can detect changes in protein synthesis in leaf tissue, plants were grown with or without cyclohexide for 4 h, and subsequently treated with 50 μM puromycin for 30 min. Using SUnSET as described above, intensity of bands representing newly synthesized proteins were inversely correlated with cycloheximide concentration, indicating that SUnSET can also be used to monitor changes in protein synthesis in leaf tissue (Fig. 1d).

**Fig. 2:**
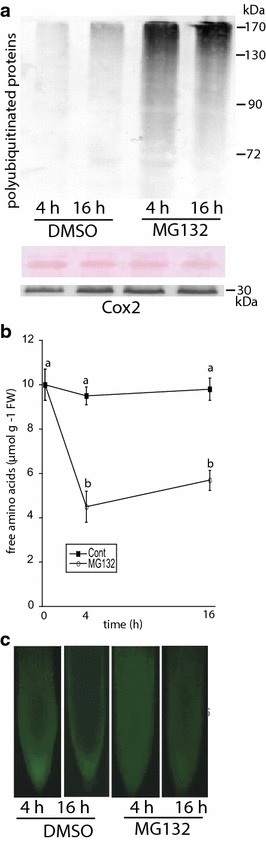
The effects of the proteasome inhibition roots treated with DMSO with or without 50 μM MG132 for 4 and 16 h. **a** Fourty μg of protein were loaded onto an 8 % SDS-PAGE gel, and levels of polyubiquitinated proteins were detected with ubitquitin antiserum. The immunoblot is representative of two other experiments. Membranes were stained with Ponceau Red prior to immunblotting. Immunoblot of Cox2 from the same membrane. **b** Levels of free amino acids in plants treated with or without MG132 for 0, 4, and 16 h. *Lowercase letters* represent a significant difference between treatments (p < 0.05, n = 5 replicates). **c** Cell viability in root tips as determined by fluorescence of fluorescein diacetate, and is representative of 20–30 roots. kDa—kilodalton

This study utilized the efficacy of SUnSET to monitor the effects of MG132—a potent proteasome inhibitor—on protein synthesis in root tissue. Initial experiments were aimed at establishing evidence that 50 μM MG132 inhibited the proteasome in the roots of Arabidopsis plants. As anticipated, proteasome inhibition caused by MG132 treatment increased levels of high molecular weight polyubiquitinated proteins after 4 and 16 h compared to control plants (Fig. 2a). MG132 treatment did not affect levels of Cox2, a subunit of the cytochrome-c oxidase complex. The proteasome degrades ubiquitinated proteins, and in yeast and animal cells its inhibition depletes the amino acid pool. Thus, it was important to determine if MG132 similarly affects amino acid levels in plants. Proteasome inhibition decreased levels of free amino acids by 55 and 43 % after 4 and 16 h, respectively (Fig. 2b). Prolonged proteasome inhibition in yeast and animal cells results in lethality, and it was desirable to determine if MG132 treatment used in this study resulted in cell death. However, MG132 did not decrease cell viability as determined by fluorescence of fluorescein diacetate, a cell permeable probe that is indicative of membrane integrity (Fig. 2c). Thus, 50 μM MG132 inhibited the proteasome, as judged by the increase in polyubiquitinated proteins and the decrease in amino acids, but did not cause lethality.

**Fig. 3:**
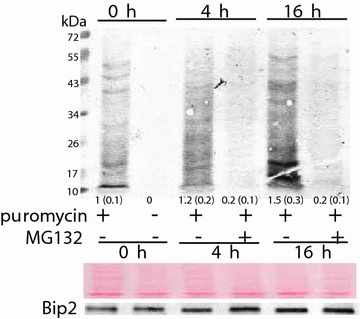
Effects of MG132 on levels of newly synthesized proteins in roots using SUnSET. Plants were treated with or without 50 μM MG132 and subsequently treated with or without 20 μM puromycin. Twenty μg of root protein were separated on a 15 % SDS-PAGE gel, and newly synthesized proteins were detected using puromycin antiserum. Membranes were stained with Ponceau Red prior to immunblotting. *Smaller letters in parenthesis* represent band intensity relative to control on dried membranes using image-J. Images are representative of four experimental replicates. Levels of Bip2 were analyzed from the same membrane. kDa—kilodalton

The effect of MG132 on protein synthesis in Arabidopsis roots was estimated by treating Arabidopsis plants with 20 μM puromycin for 30 min. Newly synthesized proteins were nearly absent in MG132-treated plants after 4 and 16 h; proteasome inhibition resulted in a faint banding pattern, but the intensity of these bands was greater compared to the negative control plants that were not treated with puromycin (Fig. 3), indicating that protein synthesis was not fully impaired by MG132. Ponceau red staining on the same membrane prior to blotting confirmed the presence of proteins. Therefore, the severe decrease in protein synthesis in MG132-treated plants could not be explained by a decrease in protein content.

**Fig. 4:**
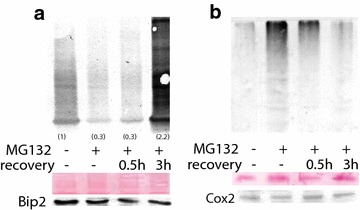
Recovery of protein synthesis in roots after removal of plants from MG132. Plants were treated with or without MG132 for 4 h, after which plants were washed and placed into Hoagland’s media without MG132 for 0.5 and 3 h. **a** Immunoreactive proteins containing puromycin were detected on a 15 % SDS-PAGE gel. *Smaller letters in parenthesis* represent band intensity relative to control on dried membranes using image-J. Images are representative of two other experimental replicates. Membranes were stained with Ponceau Red prior to immunblotting. **b** Immunoblot of polyubiquitinated proteins and Cox2

The effects of proteasome inhibition are reversible in yeast and animal cells. SUnSET was used to determine if recovery from proteasome inhibition would restore levels of newly synthesized proteins. Plants treated with MG132 for 4 h were washed and placed in media without MG132 for 0.5 and 3 h. MG132-treated plants allowed to recover for 3 h in control media had more newly synthesized proteins compared to MG132-treated plants (Fig. 4). Moreover, recovery from proteasome inhibition after 3 h resulted in higher rates of de novo protein synthesis compared to untreated plants. Increased protein synthesis after recovery was concomitant with a notable decrease in polyubiquitinated proteins, similar to control plants.

**Fig. 5:**
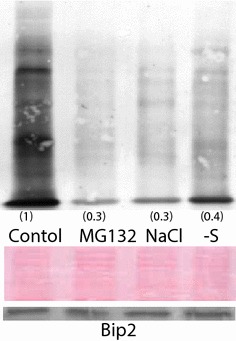
Immunoblot of newly synthesized proteins in roots using SUnSET. Plants were either untreated or treated with MG132 for 4 h, 0.1 M NaCl for 4 days, or grown under sulfur starvation for 4 days. *Smaller letters in parenthesis* represent band intensity relative to control on dried membranes using image-J. Images are representative of two other experimental replicates. Membranes were stained with Ponceau Red prior to immunblotting

In addition to proteasome inhibition, experiments were conducted to determine if SUnSET could detect changes in protein synthesis that often occur during stress. Arabidopsis plants were grown in 0.1 M NaCl or the absence of sulfur for 4 days; these stress treatments have previously been reported to decrease protein synthesis. Compared to control plants grown on complete media for an additional 4 days, salt stress and sulfur starvation decreased protein synthesis, at or near similar levels of MG132-treated plants after 4 h (Fig. 5).

## Discussion

Protein synthesis is indispensible to cellular survival, but can be altered by stress [28] and developmental patterns. Methods to measure protein synthesis in plants have traditionally required a tracer. Stable isotopes such as 15-N or 34-S can be used to gauge protein synthesis; although handling radioactivity is averted, analysis using stable isotopes is dependent upon a mass spectrometer which can be cost prohibitive. More commonly, radioactive isotopes such 35-S or 14-C are favored for both its precision and robustness, but come with risks. Recently, SUnSET techniques have been show to accurately asses protein synthesis in yeast and animal cells, and averts the health hazards associated with radioactivity.

This study represents the first time that SUnSET has been used to successfully monitor protein synthesis in plants. Therefore, this non-radioactive method is suitable to detect changes in protein synthesis in plants. This conclusion is warranted on the grounds that treatment with the protein synthesis inhibitor cycloheximide severely decreased levels of newly synthesized proteins, suggesting that puromycin only detected de novo protein synthesis in roots and leaves. Studies comparing SUnSET and radioactive methods to detect changes in protein synthesis have observed nearly identical results [21], with an r-correlation of 0.9 being reported in one study [22]. Although this study did not compare decreased protein synthesis using SUnSET and traditional radioactive techniques, the data indicate that SUnSET can be a useful method to gauge changes in protein synthesis in plants. Lastly, this study used electrophoresis to detected newly synthesized proteins; however, protein synthesis using SUnSET has also been monitored using immunohistochemistry and confocal microscopy [21, 24]. Although it is probable that protein synthesis using SUnSET can be monitored in single plant cells or intact roots via microscopy, this remains to be experimentally confirmed.

The efficacy of SUnSET was utilized to demonstrate that proteasome inhibition in Arabidopsis roots decreased levels of newly synthesized proteins, which coincided with depletion of the amino acid pool. Although these results are in agreement with other studies in yeast and mammalian cells [7], it nonetheless represents the first time that the effects of proteasome inhibition on amino acids and protein synthesis have been reported in plants. MG132 reduced protein synthesis by 70–80 %. It is possible that the deleterious effects of MG132 on protein synthesis reported in this study is underestimated, because the proteasome can remove truncated proteins containing puromycin [29].

Recently it was discovered that Arabidopsis plants initiate autophagy in response to proteasome inhibition, likely in order to replenish the amino acid pool [19]. Data presented in this study suggest that autophagy is not able to overcome the detrimental effects of MG132 treatment and restore amino acid to homeostatic levels. Although autophagy is unlikely to maintain protein synthesis during proteasome inhibition, removal of MG132 after 4-h treatment caused an accumulation of newly synthesized proteins compared to untreated plants. These results suggest that plant roots can recover from proteasome inhibition and support elevated rates of de novo protein synthesis. These results mirror the increased de novo protein synthesis in plants after a recovery from nutrient deprivation [30, 31]. A similar recovery from proteasome inhibition also occurs in neurons [32], as the removal of MG132 increased protein synthesis more than twofold compared in untreated samples. Therefore, the effects of proteasome inhibition caused by MG132 appear to be reversible in roots, as first reported in yeast [33].

To determine whether or not SUnSET provides a fast and reliable means to monitor protein synthesis during adverse environmental conditions, Arabidopsis plants were subjected to salt stress and sulfur starvation. Both of these stressors reduced levels of newly synthesized proteins, which is in agreement with other studies that examined the effects of salt stress [34] and sulfur starvation [35]. These data indicate that SUnSET is applicable to detect dramatic changes in protein synthesis, which may eventually aid both physiological and developmental studies.

## Conclusions

For the first time in plants, SUnSET was used to monitor changes in protein synthesis. As anticipated, decreased protein synthesis was observed in the roots and shoots of Arabidopsis plants treated with cycloheximide. MG132 also decreased protein synthesis in roots, but recovery from proteasome inhibition resulted in increased levels of newly synthesized proteins. Additionally, plants subjected to stress—including salt stress and sulfur starvation—also displayed a decrease in newly synthesized proteins. Collectively, these data are in good agreement with previous studies that have measured protein synthesis using radioactive materials. In this study, newly synthesized proteins were detected on a Western blot, which makes this method extremely cost effective and safer compared to methods requiring radioactive isotopes. Additionally, the protocol for SUnSET can easily be performed in <12 h. Therefore, in addition to a wide array of disciplines in plant biology, the method is suitable in educational settings where the use of radioactivity is restricted.
